# First description of the female of *Clubiona milingae* Barrion-Dupo, Barrion & Heong, 2013 (Araneae, Clubionidae)

**DOI:** 10.3897/BDJ.8.e51789

**Published:** 2020-04-24

**Authors:** Jianshuang Zhang, Hao Yu, Jian Chen

**Affiliations:** 1 School of Life Sciences, Guizhou Normal University, Guiyang, China School of Life Sciences, Guizhou Normal University Guiyang China; 2 School of Biological Sciences, Guizhou Education University, Guiyang, China School of Biological Sciences, Guizhou Education University Guiyang China; 3 Hubei Collaborative Innovation Center for Green Transformation of Bio-Resources, Centre for Behavioral Ecology and Evolution, College of Life Sciences, Hubei University, Wuhan, China Hubei Collaborative Innovation Center for Green Transformation of Bio-Resources, Centre for Behavioral Ecology and Evolution, College of Life Sciences, Hubei University Wuhan China

**Keywords:** topotype, morphology, sac spiders, taxonomy

## Abstract

**Background:**

*Clubiona milingae* Barrion-Dupo, Barrion & Heong, 2013 was described from a single male and no additional specimens have been recorded. The original description was brief and the illustrations were inadequate.

**New information:**

*Clubiona milingae* is redescribed and illustrated based on new material from the type locality and the new distribution region (Jianfeng Mountains and Limu Mountains of Hainan Island, China). The female is reported for the first time.

## Introduction

The *Clubiona apiculata* species group, ﬁrst deﬁned by Dankittipakul and Singtripop (2014) to accommodate four new species from Borneo, is one of the most distinct groups of the genus *Clubiona* sensu lato. The group presents a distinct set of characters and has been considered as putatively monophyletic (Dankittipakul and Singtripop 2014, Yu and Li 2019). The *Clubiona apiculata* group is a relatively small taxon, with only five species clearly documented, one of which is known from China (Yu and Li 2019). It is difficult to collect individuals of the *apiculata* group in the field because of their small size. At least two species have been described from only a few specimens of a single sex.

*Clubiona milingae* Barrion-Dupo, Barrion & Heong, 2013 was first described based on a single male from Mt. Jianfeng on Hainan Island, China and was not assigned to any of the existing species groups in the original publication (Barrion et al. 2013). Recently, new materials containing both sexes were collected from the type locality and near the type locality simultaneously. On the basis of the morphological characters, we matched the females and males together as *C. milingae*. Futhermore, we discovered that *C. milingae* possesses several characters associated with the *apiculata*-group and resembles *Clubiona yaoi* Yu & Li, 2019 (the only *apiculata*-group species recorded from China) due to their characteristic genital organs. Additionally, we found some characters were overlooked in the original description of the male. The aim of the current paper is to redescribe the male and report the female for the first time, providing detailed morphological descriptions and illustrations.

## Materials and methods

Spiders were fixed and preserved in 80% ethanol. Specimens were examined with an Olympus SZX7 stereomicroscope; details were studied with an Olympus CX41 compound microscope. Female epigynes and male palps were examined and illustrated after dissection. Epigynes were removed and cleared in warm lactic acid before illustration. The image of the vulva was made after being embedded in Arabic gum. Photos were made with a Cannon EOS70D digital camera mounted on an Olympus CX41 compound microscope. The digital images were taken and assembled using the Helicon focus 6.80 software package.

All measurements were obtained using an Olympus SZX7 stereomicroscope and are given in millimetres. Eye diameters were measured at the widest point. The total body length does not include the chelicerae or spinnerets. Leg lengths are given as total length (femur, patella, tibia + metatarsus, tarsus). The terminology used in the text and figure legends follows Yu and Li (2019).

All specimens are deposited in the Museum of Guizhou Education University, Guiyang, Guizhou, China (MGEU, curator Hao Yu).

## Taxon treatments

### Clubiona milingae

Barrion-Dupo, Barrion & Heong, 2013

F96C37DF-8B6C-5E0D-B74A-3B13EF724B95

#### Materials

- **Type status:** Other material. **Occurrence:** recordedBy: Qianyu Wan; individualCount: 3; sex: 2 females and 1 male; lifeStage: adult; behavior: foraging; occurrenceStatus: present; preparations: whole animal (ETOH); **Taxon:** scientificName: Clubiona milingae; acceptedNameUsage: *Clubiona milingae* Barrion-Dupo, Barrion & Heong, 2013; order: Araneae; family: Clubionidae; genus: Clubiona; specificEpithet: milingae; taxonRank: species; taxonomicStatus: accepted; **Location:** continent: Asian; island: Hainan; country: China; countryCode: CHN; stateProvince: Hainan; county: Ledong; verbatimElevation: 900-1000 m; verbatimCoordinateSystem: decimal degrees; decimalLatitude: 18.726088; decimalLongitude: 108.902750; **Identification:** identifiedBy: Hao Yu; dateIdentified: 11-2018; identificationReferences: Barrion et al. 2013; **Event:** samplingProtocol: Beating; eventDate: 10-4-2018; year: 2018; month: 4; day: 10; habitat: Rubber-tea plantation
- **Type status:** Other material. **Occurrence:** recordedBy: Jie Liu; Haiqing Ren; individualCount: 3; sex: 2 females and 1 male; lifeStage: adult; behavior: foraging; occurrenceStatus: present; preparations: whole animal (ETOH); **Taxon:** scientificName: Clubiona milingae; acceptedNameUsage: *Clubiona milingae* Barrion-Dupo, Barrion & Heong, 2013; order: Araneae; family: Clubionidae; genus: Clubiona; specificEpithet: milingae; taxonRank: species; taxonomicStatus: accepted; **Location:** continent: Asian; island: Hainan; country: China; countryCode: CHN; stateProvince: Hainan; county: Qiongzhong; verbatimElevation: 600-700 m; verbatimCoordinateSystem: decimal degrees; decimalLatitude: 19.206780; decimalLongitude: 109.768095; **Identification:** identifiedBy: Hao Yu; dateIdentified: 11-2018; identificationReferences: Barrion et al. 2013; **Event:** samplingProtocol: Pitfall trap; eventDate: 18-8-2009; year: 2009; month: 8; day: 18; habitat: Rubber-tea plantation

#### Description

**Figure 1.**
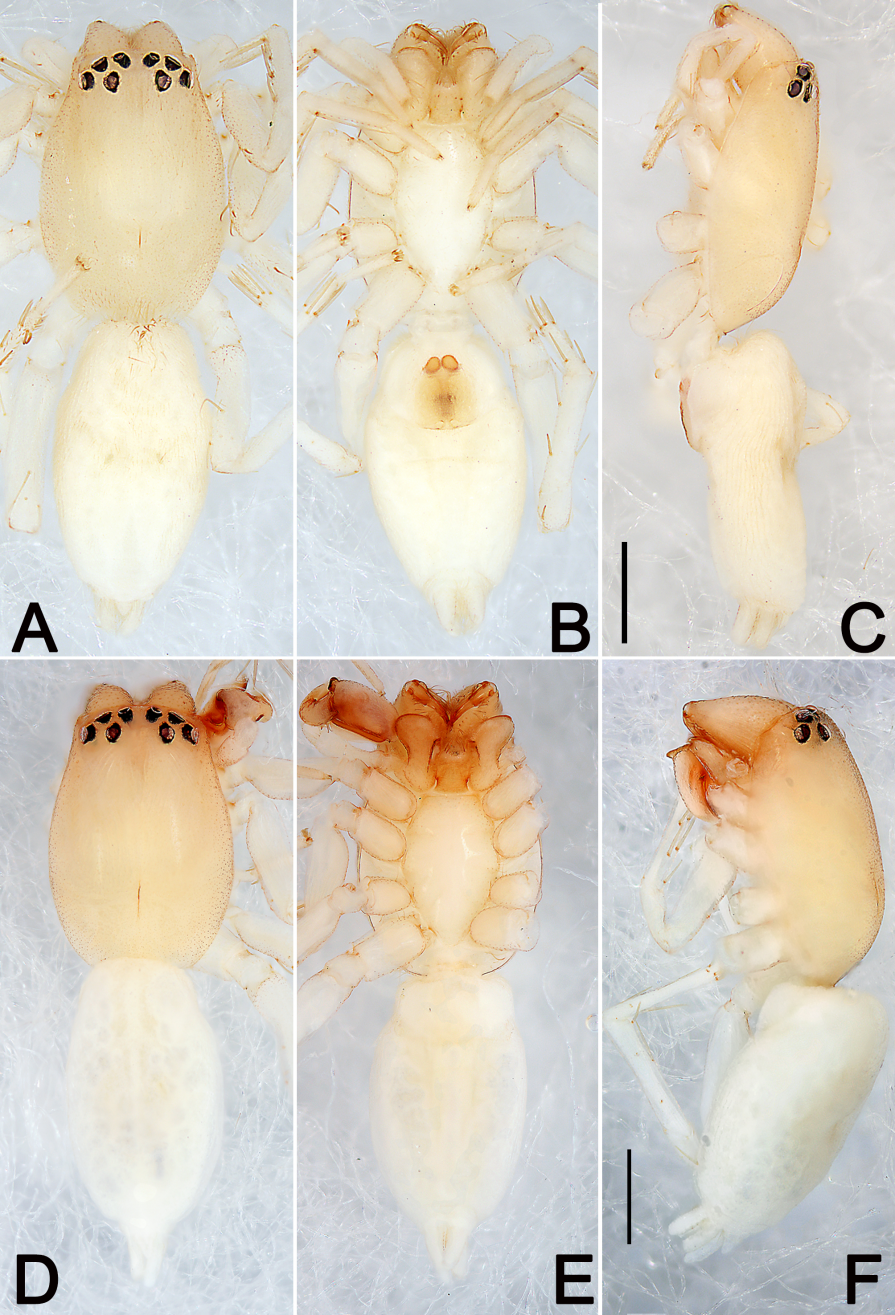
Habitus of *Clubiona milingae*, female (**A–C**) and male (**D–F**). **A, D.** Dorsal view; **B, E.** Ventral view; **C, F.** Lateral view. Scale bars: 0.5 mm (equal for **A–C**, equal for **D–F**).

***Female*** (Fig. 1A–C). Total length 2.70; carapace 1.34 long, 0.92 wide; abdomen 1.36 long, 0.78 wide.

*Carapace* yellowish-white, without distinct pattern. Fovea red. In dorsal view, anterior eye row (AER) slightly recurved, posterior eye row (PER) procurved, PER wider than AER. Eye sizes and inter-distances (mm): anterior median eyes (AME) 0.06, anterior lateral eyes (ALE) 0.08, posterior median eyes (PME) 0.09, posterior lateral eyes (PLE) 0.07; distance between AMEs (AME–AME) 0.02, distance between AME and ALE (AME–ALE) 0.04, distance between PMEs (PME–PME) 0.19, distance between PME and PLE (PME–PLE) 0.10. Length of median ocular quadrangle (MOQ) 0.20, MOQ anterior width 0.24, MOQ posterior width 0.35. *Chelicerae* coloured as carapace, with 5 teeth on promargin and 3 on retromargin. Labium and endites light brown. Sternum 0.86 long, 0.52 wide.

*Abdomen* elongate-oval, white, with inconspicuous anterior tufts of hairs, dorsum with a pair of inconspicuous muscular depressions; venter white.

*Legs* uniformly yellowish-white. Leg lengths: I 2.10 (0.64, 0.73, 0.41, 0.32), II 2.55 (0.75, 1.01, 0.45, 0.34), III 1.91 (0.66, 0.64, 0.38, 0.23), IV 3.11 (0.93, 1.08, 0.78, 0.32).

**Figure 2.**
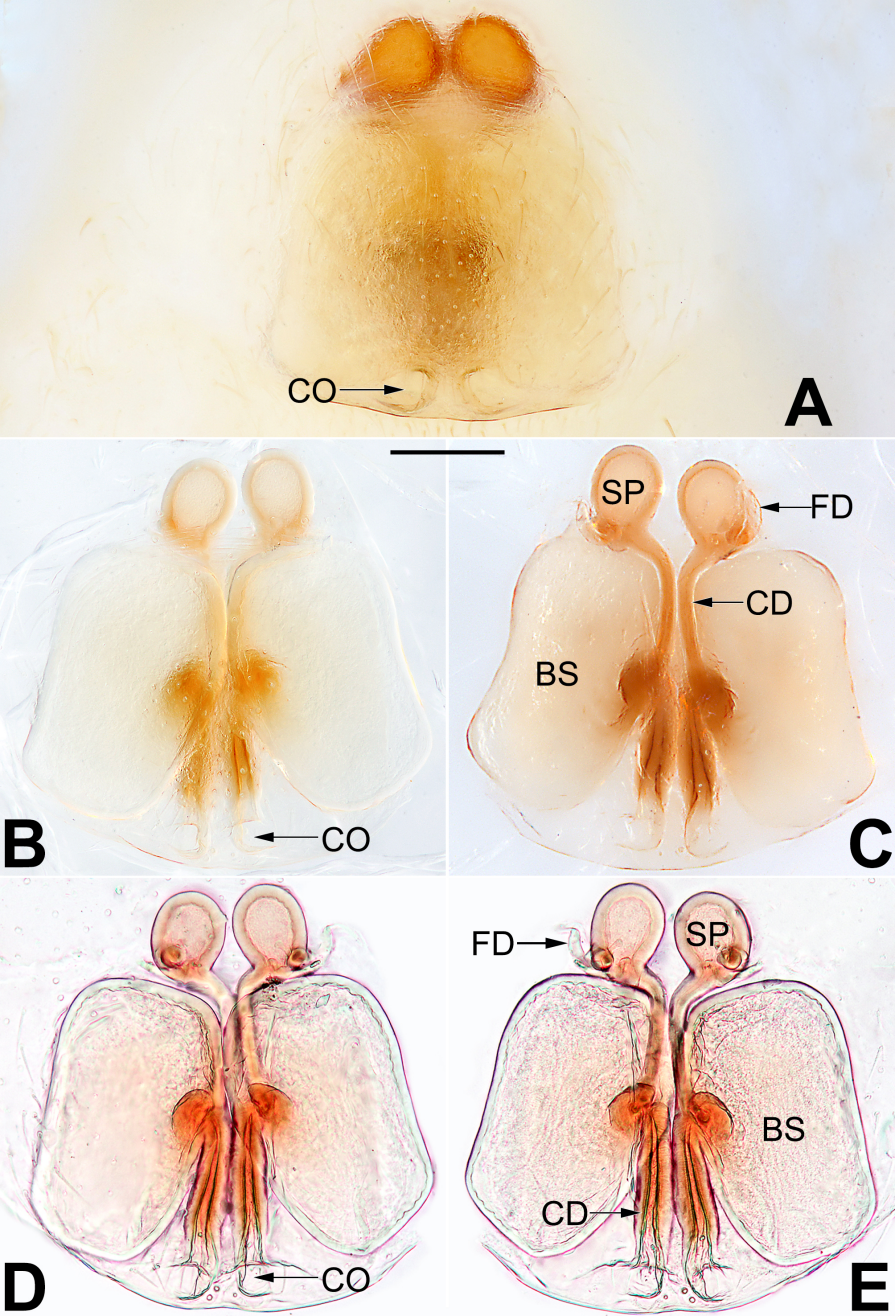
Female epigyne of *Clubiona milingae*. **A.** Epigyne, intact, ventral view; **B.** Epigyne, cleared, ventral view; **C.** Vulva, cleared, dorsal view; **D.** Epigyne, cleared and embedded in Arabic gum, ventral view; **E.** Vulva, cleared and embedded in Arabic gum, dorsal view. Scale bars: 0.1 mm. Abbreviations: BS, bursa; CD, copulatory duct; CO, copulatory opening; FD, fertilisation duct; SP, spermatheca.

*Epigyne* (Fig. 2). Epigynal plate longer than wide, anterior and posterior margin not delimited; atrium absent; spermathecae (SP) clearly visible through the tegument in ventral view; two copulatory openings (CO) small, separated by one diameter, situated at medial portion of epigynal plate posterior margin; hyaline copulatory ducts (CD) thin and straight, close together, ascending anteriorly, connected to ovoid spermathecae; bursae (BS) oblong, translucent, surface smooth, connected to copulatory ducts at mid-length between copulatory openings and spermathecae; fertilisation ducts (FD) short and curved, membranous, located on baso-dorsal surface of spermathecae.

***Male*** (Fig. 1D–F). Total length 2.64; carapace 1.46 long, 1.01 wide; abdomen 1.18 long, 0.87 wide. Eye sizes and inter-distances: AME 0.08, ALE 0.10, PME 0.09, PLE 0.09; AME–AME 0.10, AME–ALE 0.04, PME–PME 0.22, PME–PLE 0.10. MOQL 0.24, MOQA 0.26, MOQP 0.39. Sternum 0.72 long, 0.49 wide. Leg measurements: I 2.46 (0.65, 0.98, 0.44, 0.39), II 2.63 (0.79, 1.10, 0.48, 0.26), III 2.27 (0.66, 0.59, 0.73, 0.29), IV 3.18 (0.94, 1.09, 0.65, 0.50). General characters as in female but slightly smaller and darker.

**Figure 3.**
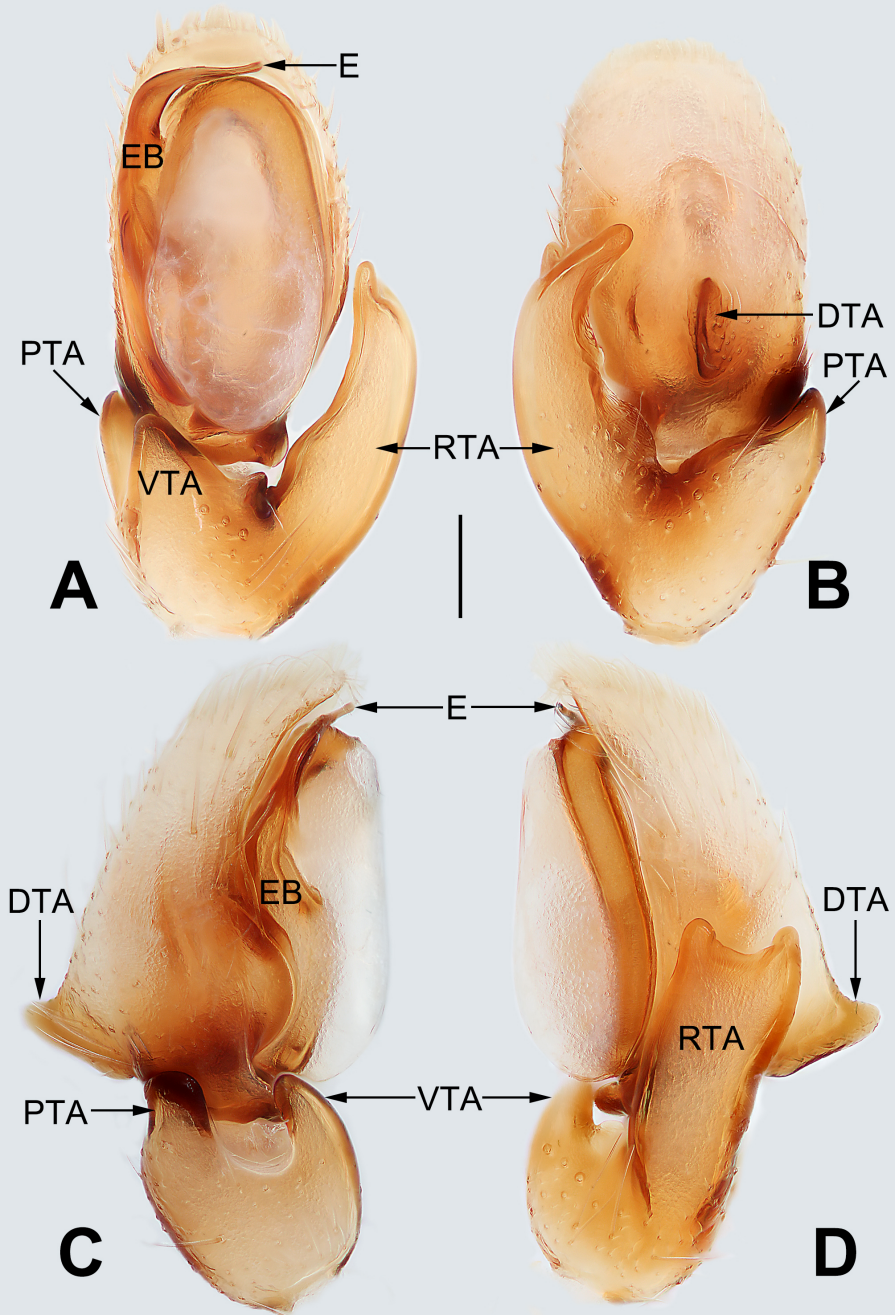
Male left palp of *Clubiona milingae*. **A.** Ventral view; **B.** Dorsal view; **C.** Prolateral view; **D.** Retrolateral view. Scale bars: 0.1 mm. Abbreviations: DCA, dorsal cymbial apophysis; E, embolus; EB, embolic base; PTA, prolateral tibial apophysis; RTA, retrolateral tibial apophysis; VTA, ventral tibial apophysis.

*Palp* (Fig. 3). Tibia short, with three apophyses, prolateral tibial apophysis (PTA) with a wide base and a blunt tip, thumb-like in prolateral view; ventral tibial apophysis (VTA) distinctly elevated, coniform in ventral view and finger-like in prolateral view; retrolateral tibial apophysis (RTA) broad and long, well-developed, tip extending to mid-length of cymbium, distally bifurcate in retrolateral view, both tips blunt, ventral ramus smaller than the dorsal one; cymbium, dorsally with cymbial apophysis, dorsal cymbial apophysis (DCA) subtriangular in lateral view; tegulum elongated and bulging, membranous and semi-transparent, except its margin in ventral view; sperm duct indistinct, starting on the apico-prolateral flank (approximately 10 o’clock of the tegulum), continuing around the tegulum, ending at embolar apex; embolus (E) relatively long, originating from retrolateral side of tegulum, its apex flagelliform and directed retrolaterally; conductor absent.

#### Diagnosis

Females of *C. milingae* can be easily distinguished from other members of the *C. apiculata*-group with the exception of *C. yaoi* (the only other *C. apiculata*-group species in China: Yu and Li 2019: 152, figures 2A–D), by the oblong bursae (bursae are spherical in all other *apiculata*-group species), but differing from *C. yaoi* by the copulatory openings situated at the medial portion of the epigynal plate posterior margin (Fig. 2A, B, D) (vs. copulatory openings situated basolaterally in *C. yaoi*). Males also resemble those of *C. yaoi* in having a retrolateral tibial apophysis with a bifurcate tip (retrolateral tibial apophysis distally unforked in all other *apiculata* -group species), but can be recognised by the relatively long embolus and by the absence of a conductor (Fig. 3A–D) (vs. embolus represented by small spicule and conductor present in *C. yaoi*). In addition, the two species can by separated by their habitus: abdomen marked with numerous inconspicuous spots in *C. milingae* (Fig. 1A, D), but with a median heart-shaped mark which extends half the length of the opisthosoma in *C. yaoi*.

#### Distribution

**Figure 4.**
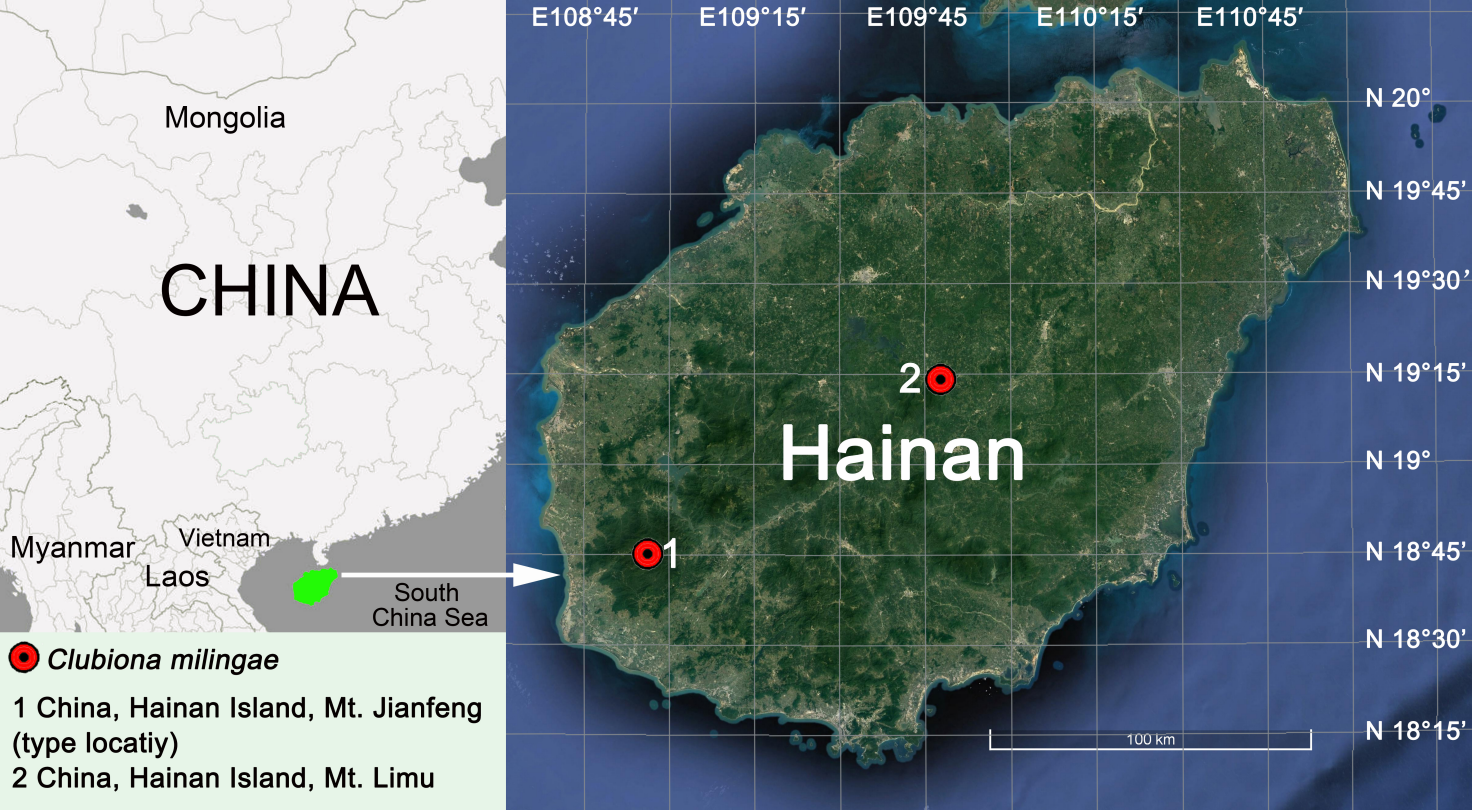
Location of Hainan Island (green) and distribution records of *Clubiona milingae* (red circles).

Known from Mt. Diaoluo and Mt. Limu, Hainan Island, China (Fig. 4).

#### Biology

Most of the new material was collected by pitfall-traps set in a rubber-tea plantation.

## Discussion

*Clubiona milingae* was first reported by Barrion et al. (2013) with a brief description and inadequate illustrations. Except for the holotype male, no additional specimens have been recorded until now. Barrion et al. (2013) did not assign the group placement of this species. Dankittipakul and Singtripop (2014) established the *apiculata* species group for four Borneo species. Maybe, due to the brief description, the inadequate illustrations, and the lack of female individuals, *Clubiona milingae* has not been assigned so far. In the present study, pairs of specimens were obtained from the type locality of *C. milingae*. The small body and widely separated PME, the presence of the apophysis on the dorsal cymbium and the strongly-developed retrolateral tibial apophysis in the male with the copulatory ducts connected to bursae at the central portion of the epigyne in the female, all indicate that this species should belong to the *apiculata*-group.

Although we have not examined the type specimen of *C. milingae*, our new topotypes bear a striking similarity to the original illustrations by Barrion et al. (2013). The slender and long, filiform embolus, the expanded RTA with a bifurcate tip, the blunt DCA and the sperm duct nearly encircling the tegulum (Barrion et al. 2013: 8, f. 7A–E) leave no doubts that our identification is correct.

## Supplementary Material

XML Treatment for Clubiona milingae
